# PD-L1 Expression Is Associated with Deficient Mismatch Repair and Poor Prognosis in Middle Eastern Colorectal Cancers

**DOI:** 10.3390/jpm11020073

**Published:** 2021-01-26

**Authors:** Abdul K. Siraj, Sandeep Kumar Parvathareddy, Padmanaban Annaiyappanaidu, Wael Haqawi, Maha Al-Rasheed, Hadeel M. AlManea, Hussah F. AlHussaini, Fouad Al-Dayel, Khawla S. Al-Kuraya

**Affiliations:** 1 Human Cancer Genomic Research, Research Center, King Faisal Specialist Hospital and Research Center, P.O. Box 3354, Riyadh 11211, Saudi Arabia; asiraj@kfshrc.edu.sa (A.K.S.); psandeepkumar@kfshrc.edu.sa (S.K.P.); pannaiyappanaidu97@kfshrc.edu.sa (P.A.); whaqawi@kfshrc.edu.sa (W.H.); mrasheed@kfshrc.edu.sa (M.A.-R.); 2 Department of Pathology, King Faisal Specialist Hospital and Research Centre, P.O. Box 3354, Riyadh 11211, Saudi Arabia; halmanea@kfshrc.edu.sa (H.M.A.); halhussaini@kfshrc.edu.sa (H.F.A.); dayelf@kfshrc.edu.sa (F.A.-D.)

**Keywords:** PD-L1, colorectal cancer, mismatch repair, prognosis

## Abstract

Several clinical trials are investigating the use of immune-targeted therapy with Programmed death ligand-1 (PD-L1) inhibitors for colorectal cancer (CRC), with promising results for patients with mismatch repair (MMR) deficiency or metastatic CRC. However, the prognostic significance of PD-L1 expression in CRC is controversial and such data are lacking in CRC from Middle Eastern ethnicity. We carried out this large retrospective study to investigate the prognostic and clinico-pathological impact of PD-L1 expression in Middle Eastern CRC using immunohistochemistry. A total of 1148 CRC were analyzed for PD-L1 expression. High PD-L1 expression was noted in 37.3% (428/1148) cases and was correlated with aggressive clinico-pathological features such as high malignancy grade (*p* < 0.0001), larger tumor size (*p* = 0.0007) and mucinous histology (*p* = 0.0005). Interestingly, PD-L1 expression was significantly higher in patients exhibiting MMR deficiency (*p* = 0.0169) and *BRAF* mutation (*p* = 0.0008). Furthermore, the expression of PD-L1 was found to be an independent marker for overall survival (HR = 1.45; 95% CI = 1.06–1.99; *p* = 0.0200). In conclusion, the results of this study indicate that PD-L1 expression could be a valid biomarker for poor prognosis in Middle Eastern CRC patients. This information can help in decision-making for anti-PD-L1 therapy in Middle Eastern CRC, especially for patients with MMR deficient tumors.

## 1. Introduction

Despite advances in cancer screening and therapy, colorectal cancer (CRC) is still a leading cause of cancer-related death worldwide [1,2]. In Saudi Arabia the incidence of CRC is increasing and it ranks as the most common cancer affecting Saudi males [3], with most patients presenting at advanced stage by the time they seek medical intervention [4]. Therefore, it is of great importance to identify new biomarkers that can predict patients’ survival and improve therapeutic options for advanced CRC patients.

One of the new emerging, promising biomarkers to evaluate patients’ outcome is programmed death ligand-1 (PD-L1). PD-L1, a transmembrane protein which binds to its inhibitory receptor PD-1 on T-cells, elicits T-cell anergy and leads to immunosuppression [5,6]. Several cancers, such as non-small cell lung cancer, melanoma, breast cancer and CRC, have been shown to upregulate surface expression of PD-L1 in order to escape the T-cell immune surveillance [7,8,9]. Monoclonal antibodies that target PD-L1 can reverse the T-cell anergy and re-sensitize tumor cells to antitumor immune surveillance [8,10]. These immunotherapies are being intensively evaluated for their efficacy in treatment of poor outcome cancers, including CRC [11,12,13]. Although the response rate to immunotherapies in unselected CRC is relatively low [14,15], PD-L1 inhibitors have shown good response in advanced CRC and some have been approved for treatment in patients with deficient mismatch repair (dMMR) refractory or metastatic CRC [16,17].

Over the past decade, expression of PD-L1 in CRC has been investigated by several studies [18,19,20,21]. The results obtained suggest that measurement of PD-L1 in CRC specimens could be useful for patients’ prognosis as well as for predicting patients that could benefit from anti-PD-L1 therapies [18,19]. However, studies analyzing PD-L1 and clinico-pathological markers and tumor prognosis are controversial. Therefore, we conducted this study using more than 1100 Middle Eastern CRCs to analyze the expression of PD-L1 and its correlation with clinico-pathological markers to explore its predictive/prognostic value in these patients.

## 2. Materials and Methods

### 2.1. Sample Selection

Archival samples from 1207 CRC patients diagnosed between 1990 to 2015 at King Faisal Specialist Hospital and Research Centre (Riyadh, Saudi Arabia) were available to be included in the study. However, immunohistochemical analysis was possible in only 1148 cases and hence these were included for further analysis. Clinico-pathological data were collected from patient medical records, which are summarized in Table 1. Institutional Review Board of King Faisal Specialist Hospital and Research Centre provided ethical approval for the current study. Research Advisory Council (RAC) granted waiver of informed consent for use of retrospective patient case data and archival tissue samples under project RAC# 2170 025.

### 2.2. DNA Isolation

DNAs were extracted from CRC tumor tissues utilizing Gentra DNA isolation kit (Gentra, Minneapolis, MN, USA) as per the manufacturer’s protocols, which were elaborated in our previous study [22].

### 2.3. Sanger Sequencing Analysis

Entire coding and splicing regions of exon 15 in *BRAF* gene among 1207 CRC samples were sequenced using Sanger sequencing technology. Primer 3 online software was utilized to design the primers (available upon request). PCR and Sanger sequencing analysis were carried out as described previously [23]. Reference sequence was downloaded from NCBI GenBank. Sequencing results were compared with the reference sequence by Mutation Surveyor V4.04 (Soft Genetics, LLC, State College, PA, USA).

### 2.4. Tissue Microarray Construction and Immunohistochemistry

Tissue microarray (TMA) format was utilized for immunohistochemical (IHC) analysis of samples. Construction of TMA was done as described previously [24]. Briefly, representative tumor regions from each donor tissue block were chosen and tissue cylinders with a diameter of 0.6 mm were punched and brought into recipient paraffin block with the help of a modified semiautomatic robotic precision instrument (Beecher Instruments, Woodland, WI, USA). Two cores of CRC were arrayed from each case.

Tissue microarray slides were processed and stained manually as described previously [25]. Primary antibody against PD-L1 (E1L3N, 1:50 dilution, pH 9.0, Cell Signaling Technology, Danvers, MA, USA) was used. A normal colon tissue microarray was also stained to validate the antibody. Normal tissues of different organ systems were also included in the TMA to serve as positive controls. Negative control was performed by omission of the primary antibody. A membranous and/or cytoplasmic staining was observed. Only the membrane staining was considered for scoring. PD-L1 was scored as described previously [26]. Briefly, the proportion of tumor cell membrane showing positive staining (brown color) was calculated as a percentage for each core. Scores from the two tissue cores of each tumor were averaged to yield a single percent staining score. For statistical analysis, the scores were dichotomized. Cases showing expression level of ≥5% were classified as over-expression and those with less than 5% as low expression.

Evaluation of mismatch repair (MMR) protein staining was performed as described previously [27]. Briefly, MMR protein expression was evaluated using MSH2, MSH6, MLH1 and PMS2 proteins. Details of the primary antibodies used are provided in Supplementary Table S1. Tumor was classified as deficient MMR (dMMR) if any of the four proteins showed loss of staining in cancer with concurrent positive staining in the nuclei of normal epithelial cells. Otherwise, they were classified as proficient MMR (pMMR).

Immunohistochemical scoring was done by two pathologists, blinded to the clinico-pathological characteristics. Discordant scores were reviewed together to achieve agreement.

### 2.5. Statistical Analysis

Associations between clinico-pathological variables and protein expression were analyzed using contingency table analysis and Chi square test. Kaplan–Meier method was used to generate overall survival curves and Mantel–Cox log rank test was used to evaluate significance. Multivariate analysis was performed using Cox proportional hazards regression model, after adjusting for clinico-pathological variables like age, gender, stage, grade, site of tumor and mismatch repair status. Two-sided tests were used for the calculations and limit of significance was defined as *p* value of < 0.05 for all analyses. Data analyses were performed using JMP11.0 (SAS Institute, Inc., Cary, NC, USA) software package.

## 3. Results

### 3.1. Patient Characteristics

The clinico-pathological characteristics of the 1148 CRC patients are summarized in Table 1. The median age of the study cohort was 56.3 years (inter quartile range (IQR), 47.5–67.9 years) with a male: female ratio of 1.1. Most of the tumors were located in the left colon (79.7%); 78.8% (905/1148) of patients had a moderately differentiated tumor and 69.9% (803/1148) were either stage II or stage III; 9.2% (106/1148) of tumors were MMR deficient by immunohistochemistry; 3.0% (34/1148) of tumors in our cohort were *BRAF* mutant. 34.6% (447/1048) patients received neoadjuvant therapy.

### 3.2. PD-L1 Expression in CRC Tissue Samples and Its Clinico-Pathological Associations

**Figure 1:**
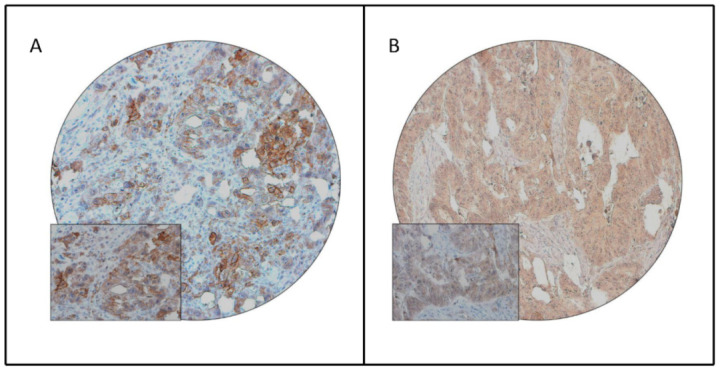
Programmed death ligand-1 (PD-L1) immunohistochemical staining in colorectal cancer (CRC) tissue microarray (TMA). Representative examples of tumors showing (**A**) positive membranous expression and (**B**) negative membranous expression of PD-L1 in CRC tissues. (20×/0.70 objective on an Olympus BX 51 microscope. (Olympus America Inc., Center Valley, PA, USA) with the inset showing a 40× 0.85 aperture magnified view of the same TMA spot). Brown color represents PD-L1 staining and blue color represents absence of PD-L1 staining.

PD-L1 protein expression was assessed immunohistochemically in 1207 CRC tissue samples. However, immunohistochemical data were interpretable in 1148 samples and hence were included for further analysis. PD-L1 membrane staining was considered for scoring (Figure 1, Supplementary Table S2). PD-L1 over-expression was noted in 37.3% (428/1148) of cases. PD-L1 expression showed significant association with high-grade (*p* < 0.0001), larger tumor size (*p* = 0.0007) and mucinous histology (*p* = 0.0005). Importantly, expression of PD-L1 was found to be significantly associated with deficient mismatch repair protein expression (*p* = 0.0169). We also found a significant association of PD-L1 expression with *BRAF* mutation (*p* = 0.0008), despite the low frequency of *BRAF* mutations in our cohort (Table 2).

### 3.3. Prognostic Significance of PD-L1 Expression in CRC

**Figure 2:**
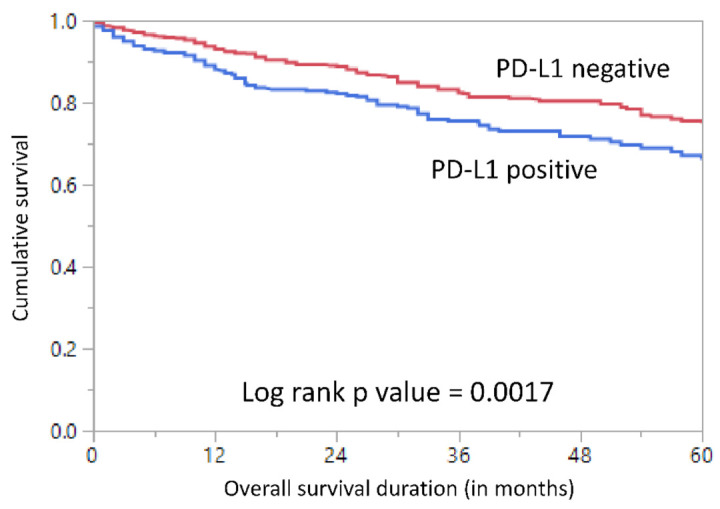
Survival analysis of PD-L1 protein expression. Kaplan–Meier survival plot showing statistically significant poor overall survival in PD-L1 positive cases compared to PD-L1 low expression (*p* = 0.0017).

PD-L1 expression was found to be associated with poor 5-year overall survival (OS) on univariate analysis (*p* = 0.0017) (Table 2, Figure 2). Next, we sought to analyze if PD-L1 was an independent prognostic predictor in CRC using Cox proportional hazards regression model, after adjusting for possible confounding factors. Multivariate analysis showed that PD-L1 expression was indeed an independent predictor of poor overall survival in our cohort (HR = 1.45; 95% CI = 1.06–1.99; *p* = 0.0200) (Table 3).

## 4. Discussion

Several studies have shown that PD-1/PD-L1 pathway has become a potential therapeutic target for a wide range of human malignancies [28,29,30,31,32,33]. However, the associations between expression of PD-L1 and clinico-pathological parameters are not consistent [18,34,35]. Tumor heterogeneity, antibody used for PD-L1 expression analysis, variation in cutoff point and sample size are among the factors which could explain the inconsistency among these studies. Therefore, in this study we comprehensively analyzed a large cohort of Middle Eastern CRC from a single institute in order to reach a better conclusion.

We found PD-L1 to be expressed in 37.3% (428/1148) of CRC tumors. There was a significant correlation between PD-L1 expression and higher tumor grade, larger tumor size and mucinous histology. Previous studies have found that PD-L1 expression correlated with advanced stage and grade [19,20,36] while other studies failed to confirm this correlation [18,37,38]. Interestingly, multivariate analysis showed that PD-L1 expression was a predictive marker for OS in the entire cohort. Analogously, a recent large meta-analysis by Wang et al. [39] in a large cohort of 8823 CRC patients from 32 studies also showed that PD-L1 expression was an independent predictor for poor OS. Another meta-analysis comprising 13 studies with 3905 CRC patients also showed a significant association between expression of PD-L1 and poor OS [40]. In contrast, a study by Li et al. [18] found PD-L1 expression in CRC tumor cells to be associated with favorable OS and disease-free survival on univariate analysis. However, this significance was lost on multivariate analysis.

Recent evidence suggests that MMR deficient tumors could provide a robust marker for anti-PD-1/PD-L1 response [16,41,42]. A strong association between PD-L1 tumor expression and MMR deficiency has been documented in endometrial and colorectal carcinoma [43,44] and the efficacy of anti-PD-1/PD-L1 therapy has been shown to increase in MMR deficient tumors [16,45,46]. MMR deficiency allows for rapid accumulation of neoantigens [47], which could explain the positive relationship between PD-L1 expression and better immunotherapeutic response in dMMR tumors. In this study, we showed significantly higher PD-L1 expression in dMMR tumors (*p* = 0.0169), suggesting that this subset of Middle Eastern CRCs may benefit from PD-L1 targeted therapy. Another mechanism of immune evasion commonly relies on mutation in *BRAF* [48]. In contrast to MMR deficiency, *BRAF* mutation in Middle Eastern CRC is not highly prevalent, with only 3% of our entire cohort being positive for this mutation (Table 1). Notably, PD-L1 expression was significantly higher in *BRAF* mutant tumors, which is in concordance with a previous study by Rosenbaum et al. [43].

However, recent evidence has shown that IHC staining of PD-L1 in CRC may not identify all patients who might respond to anti-PD-L1 agents. A phase II clinical trial (CheckMate 142) has shown similar overall response rates to immunotherapy in patients with and without PD-L1 expression [17,49]. A possible explanation could be the effect of tumor heterogeneity on the predictive value of PD-L1 expression. Indeed, this has led to the use of non-invasive molecular imaging methods (PET-CT) for PD-1/PD-L1, which are more informative, as they provide an image of the entire tumor area, both primary and metastatic, thus accounting for tumor heterogeneity [50,51,52]. However, IHC has the advantage of providing information on the cell type expressing PD-L1 (tumor cells vs. immune cells) and also being cost-effective. Hence, IHC continues to be the method of choice to determine the expression of PD-L1.

## 5. Conclusions

In conclusion, this large study from a single institute and unique ethnicity provided evidence on the importance of evaluation for PD-L1 expression and its correlation with clinico-pathological markers and prognostic outcomes. Furthermore, the information available from this report suggests that PD-L1 could be utilized as a prognostic marker in Middle Eastern CRC patients for risk stratification and that PD-1/PD-L1 inhibitors might be a valid therapeutic option for patients with advanced CRCs harboring *BRAF* mutation or showing deficient MMR. Thus, our study provides scientific rationale for clinicians to select this subset of CRC patients for anti-PD-1/PD-L1 immunotherapy. 

## Figures and Tables

**Table 1:** Clinico-pathological variables for the patient cohort (*n* = 1148).

Clinico-Pathological Parameter	*n* (%)
Age	
Median	56.3
Range (IQR)	47.5–67.9
Gender	
Male	599 (52.2)
Female	549 (47.8)
Histological subtype	
Adenocarcinoma	1019 (88.8)
Mucinous carcinoma	128 (11.2)
Histological grade	
Well differentiated	109 (9.5)
Moderately differentiated	905 (78.8)
Poorly differentiated	109 (9.5)
Unknown	25 (2.2)
Tumor site	
Left	914 (79.7)
Right	214 (18.6)
Unknown	20 (1.7)
TNM Stage	
I	148 (12.9)
II	370 (32.2)
III	433 (37.7)
IV	143 (12.5)
Unknown	54 (4.7)
MMR protein expression status	
dMMR	106 (9.2)
pMMR	1042 (90.8)
*BRAF* mutation	
Present	34 (3.0)
Absent	1092 (95.1)
Unknown	22 (1.9)
Neoadjuvant therapy	
Yes	447 (34.6)
No	701 (65.4)

IQR: inter quartile range, dMMR: deficient mismatch repair, pMMR: proficient mismatch repair.

**Table 2:** Correlation of PD-L1 IHC expression with clinico-pathological parameters in colorectal carcinoma.

Clinico-Pathological Parameters	Total		PD-L1 Positive		PD-L1 Negative		*p* Value
	*n*	%	*n*	%	*n*	%	
**Total Number of Cases**	1148		428	37.3	720	62.7	
Age (years)							
≤50	373	32.5	154	41.3	219	58.7	0.0523
>50	775	67.5	274	35.4	501	64.6	
Sex							
Male	599	52.2	217	36.2	382	63.8	0.4400
Female	549	47.8	211	38.4	338	61.6	
Tumor Site							
Left colon	914	81.0	338	37.0	576	63.0	0.6238
Right colon	214	19.0	83	38.8	131	61.2	
Histological Type							
Adenocarcinoma	1019	88.8	361	35.4	658	64.6	0.0005
Mucinous Carcinoma	128	11.2	66	51.6	62	48.4	
pT							
T1	35	3.2	11	31.4	24	68.6	0.0007
T2	169	15.5	57	33.7	112	66.3	
T3	765	70.3	264	34.5	501	65.5	
T4	119	11.0	64	53.8	55	46.2	
pN							
N0	552	50.8	192	34.8	360	65.2	0.2790
N1	332	30.6	119	35.8	213	64.2	
N2	202	18.6	83	41.1	119	58.9	
pM							
M0	955	87.0	341	35.7	614	64.3	0.1804
M1	143	13.0	59	41.3	84	58.7	
Tumor Stage							
I	148	13.5	51	34.5	97	65.5	0.4309
II	370	33.8	126	34.1	244	65.9	
III	433	39.6	162	37.4	271	62.6	
IV	143	13.1	59	41.3	84	58.7	
Differentiation							
Well differentiated	109	9.6	23	21.1	86	78.9	<0.0001
Moderate differentiated	905	80.6	324	35.8	581	64.2	
Poor differentiated	109	9.7	65	59.6	44	40.4	
MMR IHC							
dMMR	106	9.2	51	48.1	55	51.9	0.0169
pMMR	1042	90.8	377	36.2	665	63.8	
*BRAF* mutation							
Present	34	3.0	22	64.7	12	35.3	0.0008
Absent	1092	97.0	391	35.8	701	64.2	
Overall Survival							
5 Years				66.1		74.9	0.0017

dMMR: deficient mismatch repair, pMMR: proficient mismatch repair, pT: pathologic tumor size, pN: pathologic lymph node metastasis, pM: pathologic distant metastasis.

**Table 3:** Univariate and Multivariate analysis of PD-L1 expression using Cox Proportional Hazard Model.

Clinico-Pathological Parameters	Univariate		Multivariate	
	Hazard Ratio (95 % CI)	*p* Value	Hazard Ratio (95% CI)	*p* Value
Age (years) >50 (vs. ≤50)	1.06 (0.81–1.41)	0.6648	0.99 (0.72–1.38)	0.9801
Sex Male (vs Female)	1.00 (0.77–1.31)	0.9723	1.11 (0.81–1.50)	0.5183
Stage IV (vs. I–III)	5.10 (3.81–6.80)	<0.0001	5.72 (4.17–7.84)	<0.0001
Grade 3 (vs. 1–2)	1.68 (1.15–2.38)	0.0084	1.76 (1.16–2.68)	0.0083
Site Left (vs. Right)	0.77 (0.49–1.16)	0.2237	0.79 (0.50–1.25)	0.3147
MMR IHC dMMR (vs. pMMR)	0.87 (0.53–1.35)	0.5514	0.58 (0.31–0.99)	0.0483
PD-L1 expression Positive (vs. negative)	1.53 (1.17–1.99)	0.0021	1.45 (1.06–1.99)	0.0200

dMMR: deficient mismatch repair, pMMR: proficient mismatch repair.

## Data Availability

The data presented in this study are available in the article.

## Supplementary Materials

The following are available online at https://www.mdpi.com/2075-4426/11/2/73/s1, Table S1: Antibodies used for the mismatch repair immunohistochemistry assay, Table S2: PD-L1 expression stratified by proportion of immunohistochemical staining.
