# Supplementary material for: PD-L1 Expression Is Associated with Deficient Mismatch Repair and Poor Prognosis in Middle Eastern Colorectal Cancers
Source: J Pers Med. 2021 Jan 26;11(2):73. doi: 10.3390/jpm11020073 (PMC7911042; doi:10.3390/jpm11020073)
Supplement: Supplementary file 1 [file jpm-11-00073-s001.pdf]

Table S1: Antibodies used for the mismatch repair immunohistochemistry assay

Antibody	Clone	Source	Antigen retrieval	Visualization system	Dilution
MSH2	FE11	Oncogene/CalBiochem	Dako retrieval solution (pH 9)	Dako EnVision+	1:100 overnight
MSH6	44	BD Transduction Laboratories	Dako retrieval solution (pH 9)	Dako EnVision+	1:100 overnight
MLH1	G168-15	BD Pharmingen	Dako retrieval solution (pH 9)	Dako EnVision+	1:50 overnight
PMS2	C-20	Santa Cruz Biotechnology	Dako retrieval solution (pH 9)	Dako EnVision+	1:100 overnight

Table S2: PD-L1 expression stratified by proportion of immunohistochemical staining

Proportion of positively stained cells	Number of cases (%)
0%	720 (62.7)
1-20%	91 (7.9)
21-40%	102 (8.9)
41-60%	89 (7.7)
61-80%	81 (7.1)
81-100%	65 (5.7)
